# Animal models of acute exacerbation of pulmonary fibrosis

**DOI:** 10.1186/s12931-023-02595-z

**Published:** 2023-11-25

**Authors:** Xu Ye, Mingrui Zhang, Huimin Gu, Mengying Liu, Yichao Zhao, Yanchen Shi, Shufei Wu, Cheng Jiang, Xiaoling Ye, Huihui Zhu, Qi Li, Xinmei Huang, Mengshu Cao

**Affiliations:** 1 Department of Respiratory and Critical Care Medicine, Nanjing Drum Tower Hospital, Affiliated Hospital of Medical School, Nanjing University, Nanjing, Jiangsu China; 2 Department of Respiratory and Critical Care Medicine, Nanjing Drum Tower Hospital, Drum Tower Clinical Medical College, Nanjing University of Chinese Medicine, Nanjing, China; 3 Department of Respiratory and Critical Care Medicine, Nanjing Drum Tower Hospital, Drum Tower Clinical Medical College, Nanjing Medical University, Nanjing, China; 4 Nanjing Institute of Respiratory Diseases, Nanjing, China

**Keywords:** Pulmonary fibrosis, Acute exacerbation, Animal model

## Abstract

Idiopathic pulmonary fibrosis (IPF) is a chronic, progressive scarring interstitial lung disease with an unknown cause. Some patients may experience acute exacerbations (AE), which result in severe lung damage visible on imaging or through examination of tissue samples, often leading to high mortality rates. However, the etiology and pathogenesis of AE-IPF remain unclear. AE-IPF patients exhibit diffuse lung damage, apoptosis of type II alveolar epithelial cells, and an excessive inflammatory response. Establishing a reliable animal model of AE is critical for investigating the pathogenesis. Recent studies have reported a variety of animal models for AE-IPF, each with its own advantages and disadvantages. These models are usually established in mice with bleomycin-induced pulmonary fibrosis, using viruses, bacteria, small peptides, or specific drugs. In this review, we present an overview of different AE models, hoping to provide a useful resource for exploring the mechanisms and targeted therapies for AE-IPF.

## Introduction

Idiopathic pulmonary fibrosis (IPF) is a chronic, fibrosing interstitial pneumonia of unknown cause that is associated with radiological and histologic features of usual interstitial pneumonia [1]. This condition leads to a decline in physiological function, severe respiratory symptoms, and ultimately fatal clinical outcomes [2–4]. After excluding interstitial lung diseases with known etiologies, high-resolution computed tomography findings in usual interstitial pneumonia often exhibit subpleural and basal-predominant honeycombing, traction bronchiectasis, and traction bronchiolectasis [5]. These findings may coexist with ground-glass opacification and fine reticulation, which are characteristic of IPF [5]. Histopathological features of usual interstitial pneumonia include: (1) patchy dense fibrosis with architectural distortion (i.e., destructive scarring and/or honeycombing); (2) a predilection for subpleural and paraseptal lung parenchyma; (3) fibroblast foci; and (4) the absence of features that suggest an alternative diagnosis [1]. The median overall survival time of IPF patients is only 2–3 years, which is inferior to that of some cancers [6]. Moreover, a subset of patients may experience acute exacerbations (AE) of IPF (AE-IPF), which are characterized by episodic acute declines in lung function, symptoms, and quality of life [7]. Currently, pirfenidone and nintedanib are the only approved anti-fibrotic drugs used for treating IPF [8–10]. Nevertheless, these two drugs can only delay the progression of the disease and are unable to completely reverse pulmonary fibrosis [10].

To date, there is no proven effective therapy for AE-IPF [11]. A recent retrospective study of 461 patients with IPF showed that 20.8% of patients developed AE and 17.7% experienced multiple AE episodes over a median follow-up of 22.9 months [12]. A meta-analysis of six clinical trials found that the incidence of AE-IPF was 4.1% per year [13]. A Japanese cohort study revealed that the probability of AE occurrence in IPF was 8.6% at one year, 12.6% at two years, and 23.9% at three years [14, 15]. The median survival time of AE-IPF cases, without undergoing lung transplantation, was only 3–4 months [16, 17]. Notably, several factors have been consistently associated with an increased risk of AE-IPF, including the presence of pathologic fibroblastic foci and lower percentage of diffusing capacity of the lungs for carbon monoxide [18, 19]. Furthermore, elevated levels of growth differentiation factor-15, leptin, lactate dehydrogenase, Krebs von den Lungen-6, heat shock proteins, and C-reactive protein in serum have been identified as highly sensitive predictors of the incidence and poor prognosis of AE-IPF [20–27]. However, the etiology and pathogenesis of AE-IPF remain unclear, and there are no effective pharmacological treatments available except lung transplantation.

Therefore, exploring the pathogenesis and searching for therapeutic targets of AE-IPF are urgent tasks. To achieve this, reliable animal models are required. Nevertheless, while numerous compounds have demonstrated efficacy in limiting the development of pulmonary fibrosis in animal models, only a few of them have managed to replicate these beneficial effects in clinical trials [16]. It is still challenging to establish a reasonable and feasible animal model. To our knowledge, there is a lack of comprehensive summaries on animal models of acute exacerbations of pulmonary fibrosis (AE-PF), which prompted us to conduct a comprehensive analysis of recent studies on animal models of AE-PF.

### The pathogenesis of IPF

The development of IPF is a multifaceted process that involves intricate interactions between various cell types, such as type II alveolar epithelial cells (AEC2), fibroblasts, myofibroblasts, macrophages, neutrophils, and endothelial cells, as well as signaling pathways [28–32].

The convergence of advanced age, genetic predisposition, and exposure to external risk factors (e.g., smoking, occupational hazards like asbestos and silica, air pollution, viral infections, and microaspiration) can collectively lead to alveolar epithelial damage, triggering wound healing and macrophage activation [28, 33]. Persistent AEC2 injury may result in abnormal wound healing processes, epithelial-mesenchymal transition, and the release of transforming growth factor beta (TGF-β), integrin αVβ6, which promote myofibroblast activation and eventual development of pulmonary fibrosis [28]. Additionally, macrophages and neutrophils can release pro-fibrotic cytokines, including TGF-β, tumor necrosis factor-α (TNF-α), fibroblast growth factor, vascular endothelial growth factor, connective tissue growth factor, interleukin- 6 (IL-6), IL-1β, IL-8, CCL18, neutrophil extracellular traps, insulin-like growth factor-1, and platelet-derived growth factor in fibrotic niches, further stimulating fibroblast proliferation and differentiation into myofibroblasts [28, 34–38]. Ultimately, overactivation of wound healing results in the excess production of extracellular matrix due to dysregulation in the balance between matrix metalloproteinases and tissue inhibitors of metalloproteinases [10, 39].

### The pathogenesis of AE-IPF

Clinical trials published within the past decade have documented varying rates of AE-IPF, with the highest reported rate reaching 43% within a five-year period [14]. International evidence-based guidelines for IPF management tentatively recommend the use of corticosteroids during AEs and, in the long term, suggest that patients should be considered for lung transplantation [1, 14]. Therefore, it is highly significant to investigate the pathogenesis of AE-IPF.

It is currently believed that various factors, such as infection, aspiration of gastric contents, air pollution, drugs, and surgery, may trigger AE in patients with pulmonary fibrosis [14, 40–43]. Viral and bacterial infections are common culprits, with viral infections often serving as the initiating factor for AE [44, 45]. Bacteria, including both gram-positive and gram-negative bacteria, as well as fungi, can trigger AE in IPF patients. The findings of the study showed that the streptococcal species were the most common microbiota in the lung tissues of IPF patients during an episode [46]. In another survey, it was found that approximately 89.5% of bacteria in the sputum from patients with AE-IPF were Gram-negative bacteria, and Gram-positive strains only accounted for 10.5% [47]. The human herpes virus family has been extensively studied as a driver of AE-IPF [48–50]. During the COVID-19 pandemic, there have been literatures of AE induced by coronavirus infection and even vaccination [51, 52]. Additionally, studies on AE induced by the H1N1 vaccine and cytomegalovirus infections have also been reported [53–55]. Bronchoscopy or surgical procedures were frequently conducted in patients for diagnostic and treatment purposes [56]. However, both invasive procedures and direct lung surgeries can potentially trigger AE in patients with IPF [14]. Akira and colleagues reported that the occurrence rate of postoperative AE in patients undergoing lung cancer surgery with coexisting IPF ranged from 3.1–32.1% [57]. Non-pulmonary surgeries may also induce AE due to ventilator-induced barotrauma, volutrauma, or hyperoxia [14].

The apoptosis of AEC2 and excessive inflammatory/immune responses play crucial roles in the pathogenesis of infection-induced AE [47, 58]. Staphylococcus nepalensis can release corisin, a pro-apoptotic peptide that disrupts mitochondrial membrane stability. This disruption results in an increased release of pro-apoptotic factors and the inhibition of anti-apoptotic factors, ultimately promoting apoptosis in AEC2 cells and leading to AE-PF in TGF-β1 transgenic mice [59, 60]. Similarly, infection with Streptococcus pneumoniae (Spn) can lead to AE in a mouse model of lung fibrosis through the release of pneumococcal hemolysin, which induces apoptosis of AEC2 [61]. Furthermore, infection with γ-herpes virus-68 (γHV-68) increases the expression of TGF-β receptor 1 and activates TGF-β signaling in AEC2, leading to the increased phosphorylation of SMAD3 and enhanced apoptosis of AEC2 in mice with lung fibrosis, which ultimately results in the occurrence of AE [62].

In addition to enhanced AEC2 apoptosis, the dysregulation of inflammatory/immune response also plays an important factor in the pathogenesis of AE-PF. During Spn infection, glucose transporter 1-dependent glycolysis in macrophages could activate caspase-1 and triggered the production of proinflammatory cytokines such as IL-1β and IL-18, thereby promoting the exacerbation of lung fibrosis in mice [63]. Spn infection may also cause the depletion of Regulatory T cells by upregulating the response of Th1/2 cytokines (TNF-α, IL-6), leading to the deterioration of lung fibrosis in mice overexpressing TGF-β1 [37]. Moreover, McMillan TR et al [44] investigated that upregulation of chemokines (CCL2, CCL12) and subsequent recruitment of fibrocytes to the lungs during γHV-68 infection may exacerbate pulmonary fibrosis. The excessive lung inflammatory reactions induced by the proinflammatory cytokine IL-17 A and endoplasmic reticulum stress, and successfully triggered AE was observed in bleomycin (BLM) mouse model after treatment with Herpes simplex virus 1 (HSV1) [64]. Furthermore, HSV1 infection may stimulate endoplasmic reticulum stress and contribute to the expression of E3 ubiquitin ligase Ringer protein 5, leading to the deficiency of the stimulator of interferon genes and type I interferon, causing immune response disorders and ultimately resulting in acute lung injury in BLM-induced mice [65]. The Toll-like receptor 3 Leu412Phe polymorphism dysregulates the IPF lung microbiome and reduces the responses of IPF lung fibroblasts to bacterial Toll-like receptor agonists and live bacterial infection, predisposing these patients to AE-IPF [66].

**Fig. 1:**
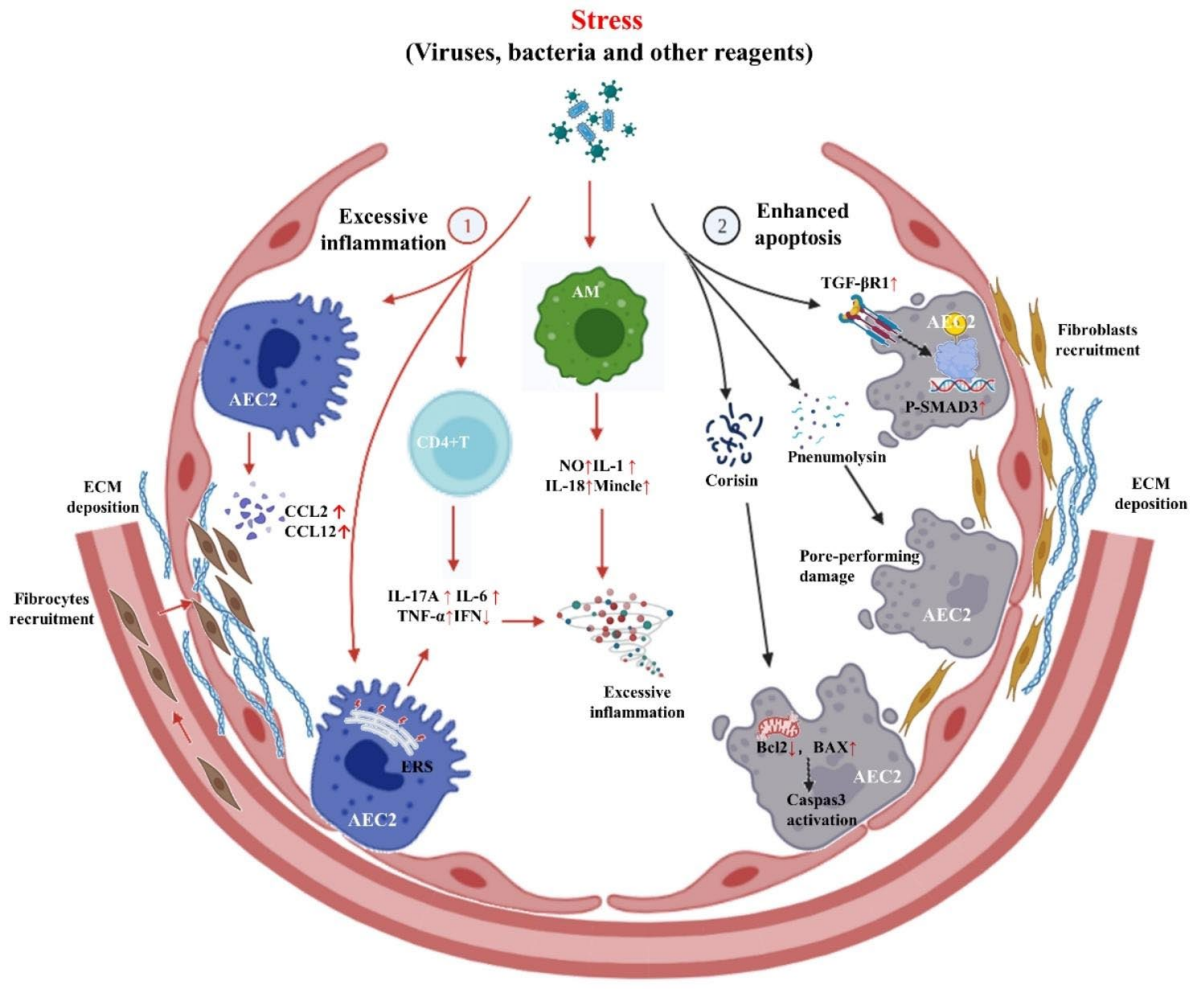
Potential mechanism of AE-PF in animal models The mechanism of AE-PF possibly driven by the stress of viruses, bacteria and other agents, such as LPS and small molecule compounds is focused on the enhanced apoptosis of AEC2, excessive inflammation and extracellular matrix deposition. Firstly, the stresses of infection and other challenging agents may promote the secretion of chemokines CCL2 and CCL12 of AEC2 and AMs, leading to increased recruitment of circulating fibrocytes and deposition of extracellular matrix. Additionally, it upregulates the expressions of pro-inflammatory cytokines TNF-α, IL-6, and IL-17 A of AEC2 and CD4^+^ T cells, while inhibits the production of IFN. Furthermore, the challenging agents can increase the expressions of IL-1, IL-18, NO, and Mincle of AMs, and lead to the excessive inflammation. Secondly, the stress may augment the expressions of TGF-β receptor 1 on AEC2, activate SMAD3 phosphorylation. Some pathogens may also produce small molecule substances which result in pore-like damage on the surface of AEC2, provoke caspase 3 activation, Bcl2 upregulation and Bax downregulation, ultimately causes the enhanced apoptosis of AEC2 **Abbreviations:** AE: acute exacerbation; AE-PF: acute exacerbations of pulmonary fibrosis; AEC2: type II alveolar epithelial cells; AMs: alveolar macrophages; BLM: bleomycin; ERS: endoplasmic reticulum stress; ECM: Extracellular matrix; HSV1: Herpes simplex virus 1; γHV-68: γ-Herpesvirus -68; LPS: lipopolysaccharide; NO: nitric oxide; Spn: Streptococcus pneumoniae; IFN: interferon

Overall, bacterial infection can activate the apoptotic pathway and induce the release of inflammatory factors, which leads to excessive inflammation and ultimately results in AE-PF. Viral infection can activate the TGF-β-SMAD3 pathway, which triggers apoptosis, or cause inflammation/immune dysregulation, fibroblast recruitment, and collagen deposition, eventually leading to aggravation of fibrosis. Lipopolysaccharide (LPS), a component of the cell wall of gram-negative bacteria, promotes nitric oxide secretion by macrophages and enhances the inflammatory response that leads to AE [67]. These underlying mechanisms of AE-PF associated with pathogenic infections and LPS stress were summarized in Fig. 1. Additionally, there are many other factors, such as the receptor for advanced glycation end product [68], adipose-derived mesenchymal stem cells [69], etc., need to be further investigated. However, given that existing models do not fully recapitulate physiologic findings in AE-IPF [48], functional studies examining host-microbiome interactions as well as better models of AE-PF are needed.

### Animal models of pulmonary fibrosis

Currently, various animal models have been utilized to investigate the mechanism of pulmonary fibrosis, including mice, rats, sheep, dogs, tree shrews, horses, donkeys, guinea pigs, and monkeys [70–75]. Although some experts have suggested that rat models exhibited a more pronounced fibrotic response than the mouse models [76], it is important to point out that a single animal model system may not fully recapitulate all aspects of the biological processes underlying human pulmonary fibrosis [16]. In 2017 official international guideline report, mice were recommended as the first-line animal model for preclinical trials, with rats being considered as a subsequent option [16].

Currently, the various mouse models of pulmonary fibrosis have been reported in the literatures, and the BLM and fluorescein isothiocyanate (FITC) are the most widely used in lung fibrosis models [70, 76]. BLM is an antibiotic produced by Streptomyces verticillus, which can induce oxidative stress response, apoptosis or necrosis, and fibrosis. These effects ultimately contribute to the development of pulmonary fibrosis by causing DNA strand breaks in cell [77]. BLM was used to induce animal fibrosis since the early 1970s [78, 79]. It is a classic challenging agent to induce pulmonary fibrosis and can be used single or multiple endotracheal, oropharyngeal, nasal, intraperitoneal, intravenous, oral inhalation, or continuous subcutaneous osmotic mini-pumps, etc [60, 70, 80]. The histological and biochemical evidences of lung fibrosis are usually noted after BLM administration for 14 days. Notably, the advantages of BLM mouse model are stability and repeatability, and Muhammad et al. introduced a multi-omics approach aimed at establishing a translational connection between a mouse model of BLM-induced pulmonary fibrosis and human IPF [80]. This model plays a crucial role in exploring the pathogenesis of pulmonary fibrosis and screening of anti-fibrosis targeted drugs, etc. However, BLM model cannot completely mimic the gradual progression and irreversibility of human IPF [81],and it lacks basal and subpleural predominance observed in human cases [28]. Interestingly, Elizabeth et al. had developed a repetitive BLM instillation approach that faithfully reproduces the histological and radiographic features observed in IPF, resulting in persistent and progressive pulmonary fibrosis in mice [82].

The induction of pulmonary fibrosis has also been associated with FITC, as it has the ability to bind to specific proteins present in lung tissue. Through the interaction of CCR2 and CCL12 ligand, FITC drives fibrocyte into the lung and induces pulmonary fibrosis. The changes of lung fibrosis are most obvious at day 21 or 28 after FITC treatment. FITC-induced model often show the site of lung injury by fluorescence signal, but cannot form fibroblastic foci, which were lack of clinical relevance and stable phenotype [81, 83]. In addition, the fibrotic response generated by different batches of FITC varies greatly as well.

Worldwide, there are an estimated 40 million workers at risk from exposure to respirable crystalline silica [84]. Exposure to inhalable crystalline silica can result in irreversible lung fibrosis, rendering it one of the methods for inducing animal models of lung fibrosis [85]. Four weeks post-intratracheal silica injection, hematoxylin-eosin staining showed alveolar structure destruction and fibrotic nodule formation. Additionally, a significant increase in the protein levels of α-smooth muscle actin (α-SMA), fibronectin, and collagen I further confirmed the successful establishment of the mouse lung fibrosis model [85]. Shen et al. administered mice with a 20µL intratracheal injection of silica suspension (250 g/L) and collected lung tissues on days 28 and 56 post-modeling. Hematoxylin-eosin staining showed increased alveolar septal thickening and a higher Ashcroft score in silicosis mice compared to saline-treated mice. Masson’s trichrome staining indicated greater collagen deposition in the silicosis group. Western blot analysis of lung lysates revealed elevated levels of collagen I and fibronectin in the silicosis group, collectively demonstrating silica-induced pulmonary fibrosis in mice [86]. Similarly, several studies have confirmed that intranasal silica instillation can induce pulmonary fibrosis in both mice and rats [87–89].

The transgenic mouse model was established for modeling pulmonary fibrosis by intratracheal delivered with adenovirus vector, followed by overexpressing of TGFα [90], TNF-α [90], TGF-β [91], IL-13 [92] or IL-1β [93]. The induction of pulmonary fibrosis was observed following targeted damage to AEC2 through the administration of diphtheria toxin in transgenic mice expressing the human diphtheria toxin receptor under the control of the AEC2 promoter region [94]. In addition, primary fibroblasts isolated from patients with IPF were injected into immunodeficient mice and a humanized mouse model of pulmonary fibrosis was successfully established [95]. Two weeks after injection of Spn intratracheally, the pulmonary fibrosis in the lung tissue were observed in miRNA-30a-5p overexpression mice, and the contents of hydroxyproline in lung tissue, the expression levels of α-SMA were significantly increased in Spn mouse model [90].

Spontaneous mouse models of lung fibrosis have been widely utilized in research. A mouse model with a mutation in the surfactant protein-C gene, specifically a cysteine-to-glycine substitution at codon 121, was created through knock-in [96]. Additionally, a knock-in mouse model was developed to allow regulated expression of an isoleucine-to-threonine substitution at codon 73 in surfactant protein-C, which is specifically linked to IPF [97, 98]. Both of the aforementioned models are capable of spontaneously developing pulmonary fibrosis. Conditional loss of Sin3a in adult mouse AEC2 leads to the development of spontaneous and progressive pulmonary fibrosis [99]. To generate Slc39a8 deletion mice, Slc39a8 floxed mice were crossbred with surfactant protein-C-CreER;Rosa26-tdTomato mice. Slc39a8 deletion mice exhibit a deficiency in the specific zinc transporter SLC39A8 within AEC2, resulting in spontaneous lung fibrosis [100]. Constructed a targeting vector with a mutation in surfactant protein A1 or put a mutation in surfactant protein A1 in the CRISPR/Cas9 to established surfactant protein A1-knock in mice, the homozygous mutant mice spontaneously developed pulmonary fibrosis [101].

There have also been reported several other fibrotic challenging agents in animal models and can cause varying degrees of lung fibrosis [70, 102, 103], such as asbestos (a single intratracheal administration), intratracheal instillation of particulate matter (PM 2.5) in mice [104], unilateral ureteral obstruction in rats [105], and chest radiation exposure [106, 107]. In addition, cyclophosphamide, methotrexate, 5‑fluorouracil, etc. have also been used to induce pulmonary fibrosis [108]. γHV-68 (1 × 10^5^ PFU) were used to induce fibrosis in aged mice (≥ 15–18 months of age) and the adenovirus injected to the trachea can cause BLM-like pulmonary fibrosis [109–111]. The rat model of lung fibrosis was established by administration amiodarone (30 mg/kg, dissolved in 0.85% normal saline, with 0.05% Tween 80) once daily by oral gavage for 3 months [112]. Nitrogen mustard was injected intratracheally into the mice by Pavel et al., and both doses (0.625 and 0.312 mg/kg, respectively) were used to induced pulmonary fibrosis in mice [113]. Moreover, a single oral gavage or intraperitoneal injection of paraquat can induce pulmonary fibrosis in rats and mice, respectively [73, 114].

## Animal models of AE-PF

### Animal models of AE-PF induced by viruses

Accumulating evidence suggests that chronic viral infections may act as persistent irritant antigens or synergistic factors in susceptible hosts to induce pulmonary fibrosis [115]. The viral RNA sequencing analysis of nasopharyngeal swabs from patients with stable IPF and AE-IPF indicated that there was a significantly higher viral positive rate in AE-IPF (60%) than in patients with stable IPF (43%). The results suggested that viral infection may be a predisposed factor for AE-IPF [49]. Hence, establishing an animal model of virus-induced AE-PF would be both reasonable and feasible.

### Herpes simplex virus 1 (HSV1) model

In this model, HSV1 (5 × 10^5^ PFU) was injected intranasally to BLM mice at Day 21 and the mice were sacrificed at Day 28 [64]. Compared to BLM animals, both the BLM and HSV1 groups exhibited higher acute lung injury scores, reduced survival rates, more severe impairment in lung function, and an excessive inflammatory response, as indicated by the increased proportion of inflammatory cells and inflammatory cytokines in bronchoalveolar lavage fluid [64]. The advantage of this model is the use of human HSV1, which can easily infect humans. Moreover, this model effectively recapitulated the progressive decline of lung function and heightened mortality rates observed in patients with AE-IPF, as well as the hallmark pathological features of acute lung injury and aberrant inflammatory signaling pathways. However, the elevation of IL-17 A and endoplasmic reticulum stress induced by HSV1 infection play a role in acute lung injury and may not be the driver of pulmonary fibrosis in mice at day 28 after BLM treatment.

AE mouse model can also be established by intranasally HSV1 (5 × 10^5^ PFU) administration on 14th day after BLM treatment [65]. The histological changes showed that the collagen deposition in the lung of BLM mice were exacerbated due to HSV1 infection. acute lung injury score, fibrosis score in mice with BLM-induced pulmonary fibrosis were increased, and the inflammatory markers such as IL-6, TNF-α and monocyte chemoattractant protein-1 in bronchoalveolar lavage fluid were also increased, while the anti-inflammatory cytokine IL-10 was decreased. Diffuse alveolar damage was characterized by interstitial edema, alveolar hemorrhage, alveolar epithelial exfoliation, and hyaline membrane formation occurred. Meanwhile, there was a reduction in forced vital capacity and lung compliance [65]. All of these findings suggested that the AEs were occurred in BLM induced pulmonary fibrosis models [65]. While the main advantage of this model was its ability to simulate some of the clinical manifestations, pathological features, and inflammatory mechanisms in AE-IPF patients, the authors did not show the hydroxyproline content, and there may be biases in the acute lung injury score and fibrosis score. Furthermore, the study did not clarify the causal relationship between endoplasmic reticulum stress and the defect of stimulator of interferon genes in AE-PF [65].

Above all, these studies demonstrated that HSV1 can induce an AE phenotype in animals with BLM-induced pulmonary fibrosis.

### γ-Herpesvirus − 68 (γHV-68) model

In order to establish AE animal model, the mice were injected murine γHV-68 (5 × 10^4^ PFU) by intranasal administration after FITC treatment for 14 days [44]. Lungs were harvested on Day 21, the excessive inflammation and collagen deposition were observed on the lung histopathology. Total lung capacity, vital capacity, and lung compliance were measured on Day 21, and the results showed that γHV-68 infection caused significant impairment of lung function in FITC-treated mice. Additionally, there was evidence of interstitial edema, intra-alveolar hemorrhage, alveolar epithelial denudation, and sloughing off of injured/dead epithelial cells [44]. The mice were treated intranasally on Day 14 with inactivated γHV-68 by exposure to ultraviolet light, on Day 21, the lungs samples were harvested, and collagen content was determined, however, there was no significant difference between mock infection and ultraviolet-inactivated γHV-68 infection. Thus, it suggested that lytic replication of the virus, not just the viral antigen, was required for the virus to aggravate FITC-induced fibrosis. While this model was able to replicate some of the clinical manifestations and pulmonary pathological changes seen in patients with AE-IPF, the mortality observed in AE mice did not increase during the 14-day period following virus treatment, unlike the high mortality of nearly 50% within a month observed in patients with AE-IPF [116]. This difference may be attributed to variations in genetics and the presence of coinfections between the animal model and the human condition. In a study by Ashley SL et al. [62] mice were treated with γHV-68 (5 × 10^4^ PFU) intranasally at 14th day in the BLM model. At Day 21, the hydroxyproline contents of lung tissues in both BLM and γHV-68 treated mice was significantly increased compared with BLM treated mice. The results of pathology showed more severe areas of excessive inflammation reactions and interstitial collagen deposition. The evidence of apoptosis of AEC2 was observed by cleaved poly-ADP ribose polymerase [62]. The main strength of this model was its ability to elucidate the underlying mechanism by which increased apoptosis of AEC2 triggers the onset of AE, a key feature of IPF. Both studies suggested that γHV-68 virus can establish AE models in BLM animals.

### Animal models of AE-PF induced by bacteria

The study showed that the lung bacterial loads in patients with AE-IPF were 4 times higher than in stable subjects. This suggests that alterations in lung bacterial loads could contribute to an increased level of lung fibrosis and the development of AE [45, 117]. As a result, bacteria are also commonly used as the triggers in animal models of AE-PF.

### Staphylococcus model

In staphylococcus models [59, 60], the combined antibiotics were used in TGF-β1 transgenic mice with pulmonary fibrosis for 4 days to eliminate the chances of pulmonary infections. On the 5th day, Staphylococcus nepalensis CNDG strain (1 × 10^8^ CFU) was injected intratracheally, and the mice were sacrificed 2 days later. Chest computed tomography (CT) and hydroxyproline of mice treated with the Staphylococcus nepalensis CNDG strain showed a significant increase in lung fibrosis and a significant neutrophil infiltration, compared with control mice treated with Staphylococcus epidermium. The increased apoptosis of AEC2 indicated that the model of Staphylococcus nepalensis CNDG strain for AE-PF was successful and feasible. In addition, TGF-β1 transgenic mice were treated with corisin, shed by Staphylococcus nepalensis CNDG, by intra-tracheal route once daily for two days before euthanasia on day 3. Compared to control mice, significantly increased infiltration of macrophages, lymphocytes and neutrophils, increased collagen deposition and concentration of inflammatory cytokines and chemokines, and enhanced apoptosis of AEC2 in the lungs were observed. This study employed a model to investigate the role of corisin, a pro-apoptotic peptide, in the development of AE in pulmonary fibrosis, and to explore its underlying molecular mechanism. Specifically, the researchers aimed to identify how corisin contributes to the onset of AE in IPF patients with bacterial infections. The study included validation experiments in both male and female mice. We eagerly anticipate the results of future studies that will build on these findings and help shed light on the complex pathogenesis of AE-IPF. While the administration of these transgenes can induce ongoing and progressive fibrosis in mice, it is important to acknowledge certain limitations of the bacterial model. These limitations include potential immune responses in the animals due to the viral vector and the expression of transgenes at levels significantly higher than what is physiologically observed [70].

### Streptococcus pneumoniae model

This AE model of mice was established by intranasally infusion with Spn strains (1 × 10^5^ CFU) at 14th day after BLM administration, compared with those treated with controls (BLM or Spn alone), the AE mice showed more severe pulmonary fibrosis and collagen deposition when sacrificed on Day 17, and the morbidity and mortality of the mice showed a dose dependence of Spn [63]. Western blot analysis showed that AIM2, activated caspase-1, and cleaved IL-1β in lung tissues were significantly increased after treatment with BLM and Spn [63]. As determined by enzyme-linked immunosorbent assay, the contents of IL-1β and IL-18 in lung tissue were significantly increased in AE mice [63]. This study revealed that activation of the AIM2 inflammasome serves as a crucial connection between glucose transporter 1-mediated glycolysis and the exacerbation of lung fibrosis induced by bacterial infection. However, the study did not report the levels of hydroxyproline in the lung tissues.

Adenovirus vector was utilized to deliver TGF-β1 to establish the model of pulmonary fibrosis in mice, and then injected Spn (1 × 10^7^ CFU) through oral-endotracheal route at 14th day [37, 61, 118]. It was proved that the intervention can successfully induce AE by observing the collagen deposition, morphological changes and Th1/Th2 cytokine levels in lung tissue. Moreover, the depleting Treg cells in the lung could promote significantly AE-PF induced by Spn. Knippenberg S et al. [61] established another mouse model which consists of diphtheria toxin administration to transgenic surfactant protein C-diphteria toxin receptor mice to cause repetitive AEC2 injury and subsequent pulmonary fibrosis, and two weeks later, the mice were treated with Spn, the content of hydroxyproline in the lung tissue of AE mice was significantly higher than that in the control mice receiving the mock infection when they were sacrificed on Day 21. The remarkable aspect of these animal models is their capacity to mimic the exaggerated inflammatory mechanisms seen in AE-IPF.

### Haemophilus influenzae model

The AE animal model with Haemophilus influenzae was also establish in BLM induced pulmonary fibrosis mice [119]. The authors administered 1 × 10^7^ CFU non-typeable Haemophilus influenzae strain NT127 intranasally to the mice on the 7th day after BLM treatment. Then the survival rate, body weight, the bacterial burden in the lung tissues and bronchoalveolar lavage fluids on the 21th day were evaluated. The results showed that the body weight and survival of mice in both BLM and NT127 group significantly decreased compared with BLM group, while the bacterial burden in the lung tissue and bronchoalveolar lavage fluids increased. The mice in both BLM and NT127 group showed more inflammatory cell infiltration in the lung tissue and severe airway structural damage than BLM group and NT127 group. This is the first report that Gram-negative bacteria have been used to induce AE model of pulmonary fibrosis. It is believed that IL-17 is secreted from γδT cells and CD4^+^ T cells, and then aggravates lung injury by the recruitment of neutrophils and eosinophils in AE model mice. While the study demonstrated increased airway inflammation in the AE group of mice, the authors did not present compelling evidence of a worsening of pulmonary interstitial fibrosis. Moreover, administering NT127 on the 7th day after BLM treatment may have been considered too early.

### Other models of AE-PF

Except for virus and bacteria were employed to induce AE occurrence in AE animal models, some biological or chemical agents such as LPS, repeated administration of BLM, cadmium chloride, and Nickel ions have been used in AE models.

LPS has been employed to induce acute lung injury in various preclinical models [120–122]. Since acute lung injury can be observed in the histopathology of patients with AE-IPF, efforts have been made to use LPS to induce AE [116]. Miyamoto H et al. [67] reported that LPS (0.05 or 0.15 mg/kg) was administered intratracheally 7 days after BLM intratracheally treated in Wistar rats. Compared with the controls, chest CT of rats in combined BLM and LPS group showed more infiltrating opacities resembling as acute respiratory distress syndrome. The histological changes showed more severe pulmonary fibrosis, and oxygen partial pressure of arterial blood decreased significantly. The plasma nitrite/nitrate, inducible nitric oxide synthase protein and mRNA levels and lung nitrotyrosine levels and the TNF-α level in bronchoalveolar lavage fluid were increased, which were similar to the pathophysiological changes of patients with AE-IPF. However, the limitations of this study are that LPS was administered on day 7, which is in the inflammatory stage rather than the fibrotic stage, and relevant indicators were monitored 24 h after LPS administration, which are different from the clinical settings. Jia K et al. [123] administered BLM intratracheal to mice on Day 1, and then LPS (1 mg/kg) was administered intratracheal on day 5, 7, and 9 to simulated AE-PF in mice. The mice in combined LPS and BLM group showed more severe inflammatory cell infiltration, thickened interalveolar space, a few collapsed alveoli in the lung tissue, a remarkable increase in collagen deposition and more severe distortion of lung architecture. Functionally, lung elastance increased and compliance and inspiratory capacity decreased. The expressions of hydroxyproline and fibrotic markers, including collagen I and α-SMA, were significantly up-regulated in mice with both BLM and LPS intervention. In particular, the expressions of inflammatory markers including IL-6, TNF-α, and IL-1β markedly increased in both BLM and LPS group compared to control group. The other study reported that LPS (0.5 mg/kg) was administered via oropharyngeal aspiration on Day 7 after BLM aspiration [124]. The changes of histological changes and CT images in the lung, lung water contents, oxygen partial pressure of arterial blood, cell counts and inflammatory cytokine levels in bronchoalveolar lavage fluid were assessed on Day 8. In both BLM and LPS group, chest CT showed diffuse ground-glass opacities, and oxygen partial pressure of arterial blood was significantly decreased. Both the BLM and LPS groups exhibited a severe inflammatory response, as evidenced by the presence of infiltrating cells in the lung histopathology and an increase in lung water content. Total cell and neutrophil counts and levels of cytokines such as monocyte chemoattractant protein-1, IL-6 and keratinocyte chemoattractant in the bronchoalveolar lavage fluid were significantly elevated in combined BLM and LPS group [124]. These models used LPS to induce AE have the advantages of excessive inflammatory responses in the lung, but lack the evidence of excessive apoptosis in AEC2. Furthermore, a single or repeated uses of LPS in the early stage is not recommended by the expert panel [16].

Repeated administration of BLM also was reported to trigger AE-PF in mice [69, 125]. The animal was injected intratracheally with BLM (4 mg/kg) to induce pulmonary fibrosis firstly, the second dose of BLM (4 mg/kg) was given on the 21th day. On Day 35, the mortality of mice and IL-6 level in bronchoalveolar lavage fluids in the two doses of BLM group were significantly higher than those in the single dose (control) group. In the AE group, the pathological changes were significantly more severe than in the control group, including the formation of hyaline membranes and the accumulation of fluid in the lungs, which are characteristic of acute lung injury in humans. The collagen scores and hydroxyproline contents in the lung and TGF-β1 level in bronchoalveolar lavage fluid were significantly higher in two doses mice than those in single-dose group. It is indicated that two doses of BLM could lead to AE of lung fibrosis. Similarly, AE model of pulmonary fibrosis was also successfully induced by a second perfusion of BLM in rats [126]. Yegen et al. established a mouse fibrosis model with four initial intratracheal injections of 0.8 UI/g BLM, given every two weeks, followed by two additional treatments of 1.6 UI/g BLM at two-week intervals to induce AE of lung fibrosis [127]. The AE group exhibited decreased body weight, higher mortality, reduced lung compliance, and significantly increased collagen synthesis compared to the BLM group. These results strongly support the successful establishment of the AE pulmonary fibrosis model [127]. An advantage of these models is their ability to corroborate the gradual progression of the disease as observed in human patients, with repeated injury and fibrosis [16]. However, the underlying assumption that fibrosis results from an external insult rather than an internal genetic/epigenetic response to injury remains unchanged [16]. Nevertheless, these models provide valuable tools for studying AE occurring in clinical settings due to non-infectious factors.

Kim MS et al. [128] established pulmonary fibrosis model in mice by tracheal instillation of polyhmethylene guanidine (PHMG, 18 µg/50µL), followed by tracheal injection of cadmium chloride (CdCl_2_, 0.2 µg/50µL) on days 3, 6, 9 and 12, respectively. Compared with PHMG group, the body weight of mice in PHMG and CdCl_2_ group was significantly reduced, the number of total cells, macrophages, neutrophils and lymphocytes in bronchoalveolar lavage fluids was increased, and collagen deposition was also increased. It was believed that PHMG and CdCl_2_ could induce the development AE-PF in mice. However, it should be noted that PHMG is not the optimal agent for inducing pulmonary fibrosis, and the timing and frequency of CdCl2 administration in this model were both too early and too frequent. Furthermore, the use of chemical reagents in this model is associated with high toxicity, rendering it unsuitable for widespread use.

The expressions of matrix metalloproteinase 9 and collagen I, inflammatory cells, the levels of IL-1β, TGF-β and TNF-α in bronchoalveolar lavage fluids were increased, while IL-10 level was decreased in BLM and nickel chloride mice compared with BLM group [129]. The hydroxyproline contents and fibrosis scores were also increased in BLM and nickel chloride group than in BLM group [129]. However, it should be noted that the simultaneous administration of nickel chloride and BLM on Day 0 in this model is not consistent with clinical practice. BLM-treated peroxiredoxin 4-transgenic mice showed more severe collagen deposition and strong immunostaining of fibronectin and worse prognosis compared to BLM-treated mice, chest CT demonstrated traction bronchiectasis and severe consolidations [130]. This finding may offer valuable insights for patients with genetically related AE-IPF.

The AE-PF animal models have provided valuable insights into disease mechanisms, but they also have certain limitations that must be addressed. While existing models can partially replicate AE features, further refinement is necessary to fully capture the complexity of this condition. To achieve a comprehensive understanding of how cell-cell interactions and soluble mediators affect AE, both biochemical and histologic analyses are crucial.

The BLM-induced mouse models produce stable and reproducible fibrotic characteristics, but it cannot mimic the progressive and irreversible nature of IPF. Recently, a strategy involving repeated BLM instillations had resulted in progressive fibrosis, recapitulating the histological and imaging characteristics of IPF. This approach offers insights into addressing this issue [82]. On the other hand, the FITC-induced lung fibrosis model accurately displays lesions through fluorescence signals, but lack of clinical relevance and inability to form fibroblastic foci limit its use. In addition, the TGF-β transgenic model induces sufficient fibrosis, but overexpression of transgenes may result in excessive fibrosis, and mice may develop an immune response to adenoviral vectors. Simulating the pathogenesis of IPF by damaging AEC2 is a cumbersome process, and repeated drug use may lead to drug resistance. Moreover, the toxic nature of reagents used in this approach poses risks to laboratory personnel. Although using viruses or bacteria to induce AE can accurately simulate clinical settings, some pathogens may only exacerbate inflammation and not fibrosis. Chemical reagents can be used to induce AE in mouse models, but timing and frequency of administration must be optimized, and the toxicity of certain reagents limits their widespread use. Despite these limitations, animal models provide a valuable tool for understanding the pathogenesis of AE-IPF and identifying potential therapeutic targets. With continued refinement and validation, these models may offer a promising avenue for advancing our understanding of this devastating disease.

Taking into account the advantages and disadvantages of established animal models, researchers are actively exploring the development of novel animal models. Pigs, due to their anatomical size and structure, physiology, immunology, and genome similarity to humans, have been utilized in the investigation of various lung diseases [131, 132]. A model of unilateral acute lung injury in pigs has been established, validated by histopathology and an inflammatory response [133]. This model can serve as a basis for comparing the pathophysiological changes in both the injured and uninjured lungs within the same animal [133], and holds potential for the study of AE-IPF in the future. Numerous studies have explored the utilization of sheep as an animal model for pulmonary fibrosis. Organ et al. employed a bronchoscope to inject BLM into the lung base, effectively replicating the clinical characteristics of IPF, which often manifest as subpleural and basal-predominant [134]. The study demonstrated a correlation between fibrosis and impaired lung function for up to seven weeks following BLM treatment [134]. Leveraging the larger size of sheep, multiple blood gas analyses were conducted, facilitating the monitoring of oxygen and carbon dioxide partial pressures [135]. This approach accurately simulates clinical practice for IPF patients who experience severe hypoxia and necessitate dynamic blood gas monitoring. In addition, Perera et al. successfully reproduced factors that may be involved in the pathogenesis of IPF, such as ER stress and AEC2 and macrophage apoptosis [136]. Importantly, their research indicated that administering calcium-activated potassium channel inhibitors in vivo could alleviate endoplasmic reticulum stress and the apoptosis of AEC2 and macrophages [136]. Furthermore, significant miRNA expression similarities between sheep models and IPF patients were revealed by database analysis, in contrast to mice [137]. This valuable information contributes significantly to the study of IPF pathogenesis and the quest for therapeutic targets.

Furthermore, since chest imaging and pulmonary function indicators are often the primary endpoint in clinical trials [70], these parameters warrant further investigation in future animal experiments. In upcoming investigations, some novel peripheral blood biomarkers may have wider applications. Among these are particular sections of type IV collagen, such as a1 (C4M12a1) and a3 (C4M12a3) chains, as well as neoantigens generated by the cleavage of matrix metalloproteinases [16].

Lung slice cultures offer several benefits, including the identification of new targets, the facilitation of kinetic investigations, the comparison of biological processes between human and murine systems, and the examination of slices from IPF-affected lungs [16]. However, precision cut lung tissues have a significant limitation in that they have a short lifespan of approximately one week, which makes them unsuitable for use as chronic, long-term drug testing platforms [138].

**Fig. 2:**
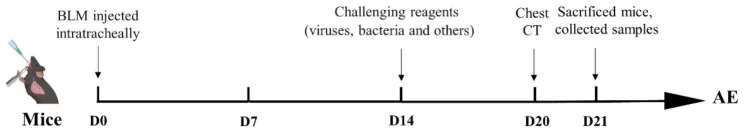
Schematic diagram depicting the mouse model of AE-PF On Day 0, mice were administered with a single dose of BLM via the transtracheal route. On Day 14 after BLM treatment, mice in the AE group were challenged with bacteria, viruses, or other challenging reagents such as BLM or lipopolysaccharide. Chest CT scans were performed on Day 20 before animal sacrificed. The mice were killed on Day 21, and the samples of blood, bronchoalveolar lavage fluids, and lung tissues were collected Abbreviations: AE: acute exacerbation; AE-PF: acute exacerbation of pulmonary fibrosis; BLM: bleomycin; CT: computed tomography

We summarize the currently published AE animal models of pulmonary fibrosis in Table 1. Based on the data, there is considerable inconsistency in time points of triggers to induce AE. As we know, the administration of BLM is the commonly used experimental system to study IPF pathogenesis [103, 139]. This dosing regimen transforms into a fibrotic response from approximately day 14, and may spontaneously resolve beyond 28 days [70]. For an accurate evaluation of anti-fibrotic efficacy, the intervention should target fibrosis [139]. Similarly, to accurately assess the effect of challenging agents for AE and study pathogenesis of AE-PF, we propose an ideal AE model should be performed in the fibrotic phase (on the 14th day after BLM administration) and collect the samples of blood, bronchoalveolar lavage fluid and lung tissues at Day 21 to evaluate whether AE occurred and the possible mechanism (shown in Fig. 2).

**Table 1:** Animal models of AE-PF

Animal species	Fibrotic Agents	Challenging agents of AE				Lung pathology	Advantages	Disadvantages	References
		Agent	Dose	Route	Time of administration				
Mouse	FITC	γHV-68^①^	5 × 10^4^ PFU	I.N	D14	Interstitial edema, intraalveolar hemorrhage, alveolar epithelial denudation.	Introduced a novel animal model for herpesvirus-induced AE-PF and offered insights into the underlying mechanisms, including fibrocyte recruitment.	The mortality rate in AE mice did not align consistently with that observed in AE patients.	McMillan et al., 2008 [44]
Mouse	BLM	γHV-68^①^	5 × 10^4^ PFU	I.N	D14	Focal mononuclear inflammation, diffuse inflammation, stromal collagen deposition.	The model elucidated the underlying mechanism by which increased apoptosis of AEC2 triggers the onset of AE, a key feature of IPF.	The evidence of apoptosis measured by cleaved poly-ADP ribose polymerase was inconclusive.	Ashley et al., 2014 [62]
Mouse	SPC-DTR	Spn^②^	1 × 10^7^ CFU	O.T	D14	---	The model mimicked the exaggerated inflammatory mechanisms seen in AE-IPF.	The establishment of the SPC-DTR fibrosis model requires repeated administration, which may lead to drug resistance.	Knippenberg et al., 2015 [61]
Mouse	BLM	LPS^③^	0.5 mg/kg	O.A	D7	Fibrotic changes, prominent rise in inflammatory cell infiltration.	Assessing the results using chest CT scans and blood gas analysis is more clinically relevant.	Operation-induced AE-IPF occurs rarely in patients, making the practicality of this model poor.	Kimura et al., 2015 [124]
Mouse	BLM	BLM^③^	4 mg/kg	I.T	D21	Hyaline membrane formation, pulmonary edema, a large amount of collagen deposition	The mouse model of AE-IPF was triggered by a non-infectious mechanism.	The core assumption that fibrosis stems from an external trigger rather than an internal genetic or epigenetic response remains unchanged	Wei et al., 2016^127^
Mouse	BLM	HSV1^①^	5 × 10^5^ PFU	I.N	D14	Diffuse alveolar damage, interstitial edema, intraalveolar hemorrhage, alveolar epithelial denudation, hyaline membranes formation, collagen deposition.	This model simulated some of the clinical manifestations, pathological features, and inflammatory mechanisms in AE-IPF patients.	The causal association between endoplasmic reticulum stress and stimulator of interferon genes deficiency needs further investigation.	Qiu et al., 2017 [65]
Mouse	PHMG	CdCl2^③^	0.2 µg	I.T	D3, D6, D9, D12	Granulomatous inflammation, fibrosis alveolar ducts, foamy macrophage infiltration, bronchioloalveolar epithelial hyperplasia, collagen deposition.	Because CdCl2 is a component of tobacco, this model simulates lung fibrosis that may be caused by tobacco.	The reagents are toxic, and PHMG is not the preferred agent; the timing of CdCl2 administration was too early.	Kim et al. 2018 [128]
Mouse	BLM	Nickel chloride^③^	5 mg/kg	I.T	D0	Collagen deposition, alveolitis, collapsed alveolar space, inflammatory cells accumulation.	Nickel is heavy metal that can simulate lung fibrosis caused by industrial pollution.	Nickel chloride and BLM were administered simultaneously, which is not consistent with clinical settings.	McElroy et al., 2018 [129]
Mouse	BLM	HSV1^①^	5 × 10^5^ PFU	I.N	D21	Alveolar septal congestion, edema, inflammation, alveolar epithelial damage, hyaline membrane formation.	This model uses human HSV1, mirroring AE-IPF symptoms including lung function decline, increased mortality, acute lung injury, and abnormal inflammation pathways.	The elevated levels of IL-17 A and endoplasmic reticulum stress may not be the primary drivers of pulmonary fibrosis.	Chen et al., 2019 [64]
Mouse	Ad TGF-β1	CNDG^②^	1 × 10^8^ CFU	I.T	D5	Significantly increased macrophage, lymphocyte, and neutrophil infiltration, enhanced collagen deposition, increased epithelial cell apoptosis.	The study included validation experiments in both male and female mice.	Animals may mount an immune response; the expression of transgenes may exceed physiological levels.	D’Alessandro-Gabazza et al., 2020 [59]
Mouse	BLM	Spn^②^	1 × 10^5^ CFU	I.T	D14	A significant increase in fibrosis and collagen deposition	This study found that AIM2 inflammasome activation plays a crucial role in worsening lung fibrosis during bacterial infections by connecting it to glucose transporter 1-mediated glycolysis.	Did not report the levels of hydroxyproline in the lung tissues	Cho et al., 2020 [63]
Mouse	Ad TGF-β1	Spn^②^	1 × 10^7^ CFU	O.T	D14	Increased interstitial inflammation, alveolar epithelial hyperplasia, thickened alveolar septa, elevated collagen deposition	This study enhances our understanding of infection-induced lung fibrosis exacerbation in two preclinical mouse models.	Transgene expression was higher than physiologically possible; Possible immune response to viral vectors.	Moyé et al., 2020 [37], Knippenberg et al., 2015 [61]
Mouse	BLM	Corisin^③^	300 µg	I.T	D20	Significantly increased Ashcroft score and collagen-stained (trichrome) area.	The study included validation experiments in both male and female mice.	Future studies should assess corisin’s efficacy in inducing AE in various mouse models of pulmonary fibrosis.	D’Alessandro-Gabazza et al., 2022 [60]
Mouse	BLM	NT127^②^	1 × 10^7^ CFU	I.T	D7	Increased inflammatory cell infiltration, airway structural damage, collagen deposition.	This is the first report that Gram-negative bacteria have been used to induce AE model of pulmonary fibrosis.	NT127 administrated on day 7 was not in the fibrotic stage; no further evidence of increased interstitial inflammation	Chen et al., 2022 [119]
Mouse	BLM	LPS^③^	1 mg/kg	I.T	D5, D7, D9	Severe inflammation, thicker interalveolar spaces, collapsed alveoli, increased collagen, and severe lung distortion.	Simulates extreme inflammation in AE-IPF.	LPS-induced AE was applied on days 5, 7, and 9 during the inflammatory phase, rather than the fibrotic phase.	Jia et al., 2022 [123]
Rat	BLM	LPS^③^	0.05 or 0.15 mg/kg	I.T	D7	Significant fibrosis, prominent inflammatory cells infiltration and alveolar enlargement	Assessing the results using chest CT scans and blood gas analysis is more clinically relevant. Simulates extreme inflammation in AE-IPF.	LPS-induced AE was monitored at 24 h after administration, which differs from clinical settings where changes in related indicators may occur later.	Miyamoto et al., 2022 [67]

**Abbreviations**: AE: acute exacerbation; AE-PF: acute exacerbation of pulmonary fibrosis; Ad TGF-β1: adenoviral vector delivery of active transforming growth factor-β1; BLM: bleomycin;CdCl2: cadmium chloride; CNDG: staphylococcus nepalensis strain CNDG; FITC: fluorescein isothiocyanate; HSV1: Herpes Simplex virus 1; γHV-68: γ-Herpesvirus − 68; I.N: intranasally; I.T: intratracheally; LPS: lipopolysaccharide; NT127: non-typeable Haemophilus influenzae strain 127; O.T: orotracheally; O.A: oropharyngeal aspiration; PHMG: polyhmethylene guanidine; SPC-DTR: surfactant protein C-diphteria toxin receptor

① .Virus; ②.Bacteria; ③.Others

## Conclusion

Establishing a consistent and reliable animal model of AE-PF holds great significance in understanding its pathogenesis. We have summarized the various animal types, inducers, and dosages employed in existing models, with bleomycin and mice continuing to be the most frequently utilized choices. Each model exhibits its unique attributes, and the timing of interventions plays a vital role in the development of an effective AE-PF model. Administering challenging agents for AE at the peak level of fibrosis would be the ideal approach. Although these animal models may not replicate the exact characteristics of AE-IPF patients, they nevertheless provide valuable insights into the pathobiology associated with cells, mediators, and signaling pathways that may be involved in fibrosis as well as AEs. Additionally, the AE animal model of pulmonary fibrosis serves as a crucial means to evaluate hypotheses regarding novel therapies. Hopefully, establishing an ideal animal model of AE-PF will provide an important tool for exploring the mechanisms underlying this debilitating disease and identifying potential therapeutic targets.

## Data Availability

Not applicable.

## Abbreviations

AE: acute exacerbation
AE-PF: acute exacerbations of pulmonary fibrosis
AEC2: type II alveolar epithelial cells
BLM: bleomycin
FITC: fluorescein isothiocyanate
HSV1: herpes simplex virus 1
IPF: idiopathic pulmonary fibrosis
γHV-68: γ-Herpesvirus − 68
α-SMA: α-smooth muscle actin
LPS: lipopolysaccharide
Spn: streptococcus pneumoniae
TGF-β: transforming growth factor β
TNF-α: tumor necrosis factor α
